# Bioinspired superhydrophobic surfaces, fabricated through simple and scalable roll-to-roll processing

**DOI:** 10.1038/srep15430

**Published:** 2015-10-22

**Authors:** Sung-Hoon Park, Sangeui Lee, David Moreira, Prabhakar R. Bandaru, InTaek Han, Dong-Jin Yun

**Affiliations:** 1 Department of Mechanical engineering, Soongsil University, 369 Sangdo-ro, Dongjak-gu, Seoul, 156-743, Korea; 2 Material Research Center, Samsung Advanced Institute of Technology, Yongin-si, Gyeonggi-do, 446-712, Korea; 3 Department of Mechanical & Aerospace Engineering, University of California,San Diego, La Jolla, CA 92093-0411, USA

## Abstract

A simple, scalable, non-lithographic, technique for fabricating durable superhydrophobic (SH) surfaces, based on the fingering instabilities associated with non-Newtonian flow and shear tearing, has been developed. The high viscosity of the nanotube/elastomer paste has been exploited for the fabrication. The fabricated SH surfaces had the appearance of bristled shark skin and were robust with respect to mechanical forces. While flow instability is regarded as adverse to roll-coating processes for fabricating uniform films, we especially use the effect to create the SH surface. Along with their durability and self-cleaning capabilities, we have demonstrated drag reduction effects of the fabricated films through dynamic flow measurements.

Superhydrophobic (SH) surfaces, *i.e.* surfaces with a water contact angle (WCA) above 150° and a hysteresis angle lower than 5°, have are of extensive interest in various scientific and engineering fields^1^,^2^, with various applications including self-cleaning surfaces, anti-icing coatings, anti-adhesion coatings, and microfluidic systems^3^,^4^,^5^,^6^,^7^,^8^. Such surfaces are typically created by coating substrates with low-surface–energy materials coupled with controlling the surface roughness at both the micro- and nano-scales^9^.

While a variety of approaches to SH behavior have been developed^10^,^11^,^12^,^13^,^14^, there are still critical barriers to their widespread use due to the cost, number of processing steps, limits on the manufacturable area, durability etc. For example, a soft-lithographic imprinting method using a polydimethylsiloxane (PDMS) stamp, which was prepared by replica molding against a hydrophobic lotus-leaf surface, was developed for SH surfaces^15^. Using such processes, the highest WCAs were achieved when patterns with high aspect ratio were used. However, such patterns were easily damaged through tribological interactions. Recently, designs incorporating a dual-hole pattern and a hierarchical micro-/nano-structure were developed for robustness against tribological damage^16^,^17^. However, such methods involved require a complicated series of steps and are consequently limited to small area substrates. With proper processing temperatures, solvent-casting methods could also be used to form SH coatings on various substrates^18^. Furthermore, in conjunction with conjugated conducting polymers, such methods could also be used to create electrically active SH coatings. However, durability and uniformity yet remain key issues restricting commercial application. In other approaches, nanoparticle/fiber coatings on textiles have produced robust SH surfaces; this is possibly the method most amenable to practical applications^19^,^20^,^21^, but such methods are not applicable in all situations due to the requirement that the fabric have microscale roughness^22^. Additionally, laminate-templating methods using a membrane or mesh plate were developed to produce SH surfaces with abrasion resistance by laminating thermoplastic film and microscale mesh template^23^,^24^,^25^, heating under pressure to near the film’s melting temperature, cooling, and then peeling off the template. Hair-like SH surface structures were developed through the capillary effects between the mesh and film. However, such methods are limited yet by the heating cycles, the production time and issues related to the template reusability.

Through a careful consideration of such state-of-the art techniques, we developed a simple as well as scalable roll-coating based process for fabricating SH surfaces harnessing non-Newtonian flow instabilities and associated effects, such as ribbing^26^,^27^,^28^,^29^,^30^. Our method has the advantage of requiring no additional chemical, vacuum, heat-treatment, or cleaning steps, nor any patterned template such as a master stamp or mesh. In addition to the efficiency and scalability of the process, the resulting tribologically stable SH surface has a bristled shark-skin like structure which could facilitate drag reduction effects in dynamic liquid flow over the surface.

## Results

### Formation of the bristled shark-skin like SH pattern

**Figure 1:**
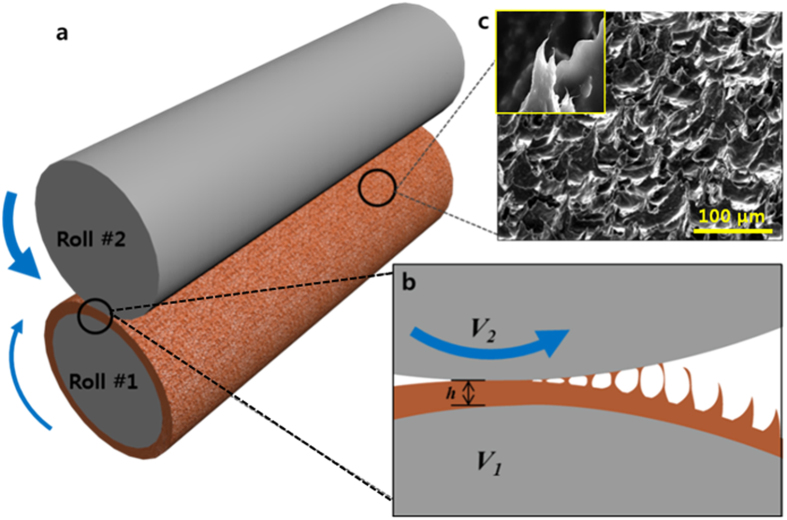
Fabrication of the shark-skin–like patterned films. (**a**) Schematic diagram of the double-roll film-making machine. (**b**) Pattern formation results when capillary bridges form between the rolls when their rotation speeds are inverted. (**c**) SEM images of the pattern produced by the rolling process. In inset image, individual MWCNTs were observed at the end of the pattern tip.

A double-roll based process was used to fabricate the SH film, as schematically shown in Fig. 1a. More detailed images of the apparatus/machine are shown in the Supplementary Information (Supplementary Fig. 1). A high viscosity paste (comprised of 10 wt% multiwalled carbon nanotubes (MWCNTs) dispersed into polydimethylsiloxane (PDMS)) was placed in between the rollers, and the paste was transferred onto the roll with the higher speed, *e.g.,* roll #1 (rotating at a velocity of *V*_1_) and roll #2 (rotating at a velocity of *V*_2_). Where if *V*_1_ > *V*_2_, the paste was coated as a smooth film (with less than 300nm roughness) onto roll #1 (as shown in Supplementary Fig. 1c).

When *V*_2_ exceeds *V*_1_, it was observed that the paste on roll #1 starts to stick and then transfer to roll #2 in the nip area between the two rolls by capillary bridging^31^, as shown Fig. 1b. At a certain *V*_*2*_*/V*_*1*_ ratio (>1), a randomly structured pattern develops over the entire film area as indicated in Fig. 1c. Additionally, individual MWCNTs were observed at the end of the pattern tip due to capillary bridge effects. The resulting pattern, consisting of micro- and nano-scale features, resembles that of bristled shark skin with comparable scales and suggests possible use for a low drag material^32^,^33^. More detailed figures are shown Supplementary Fig. 2, demonstrating the large-scale uniformity of the films.

**Figure 2:**
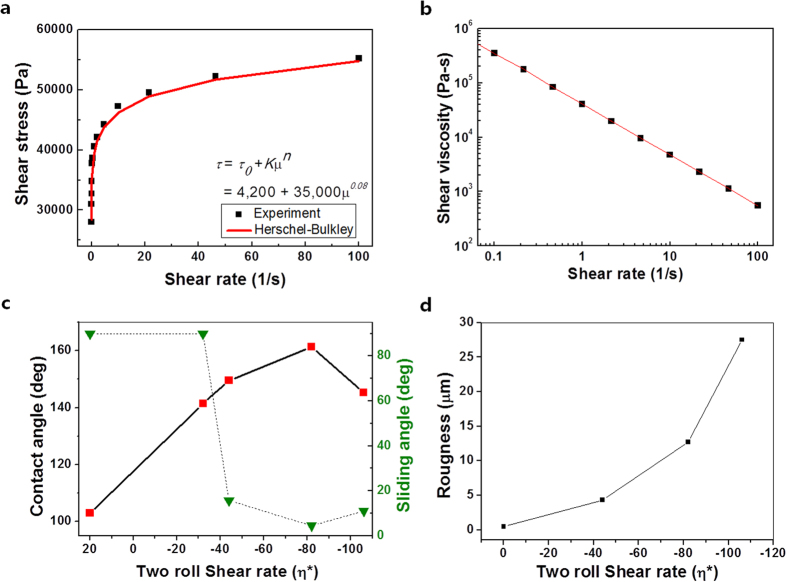
Properties of the high viscosity MWCNT paste and the developed patterns. (**a**) Shear stress and (**b**) shear viscosity of the 10 wt% MWCNT/PDMS paste at various shear rates. (**c**) WCA and SA properties of the patterns generated at different roll shear rates (*η*). (d) Roughness of the patterns generated at different roll shear rates.

High shear/yield stress (*τ*_0_) and thixotropic viscosities, important for maintaining the pattern structure, were measured (10 wt% MWCNT paste), as indicated in Fig. 2a,b^26^,^34^,^35^,^36^. For the fabrication of high-viscosity paste, three-roll milling techniques were used to disentangle and disperse high aspect ratio MWCNTs in the polymer matrix^37^. The paste was under shear force in the nip region between the roller contact area (Fig. 1b). Then, the paste was not sheared after rolling out from the nip between the rollers, which means the shear rate was nearly zero, and the developed morphology was unchanged by the yield stress. The non-Newtonian shear stress and viscosity of the MWCNT paste were shown to obey the Herschel-Bulkley equation^35^.




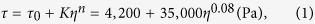




where, *τ*_0_ is the yield stress, *η* is the shear rate (1/sec), and *K* and *n* are constants. The value of n was less than 1.0, which implies the MWCNT/PDMS paste has a shear-thinning behavior, shown in Fig. 2b.

**Figure 3:**
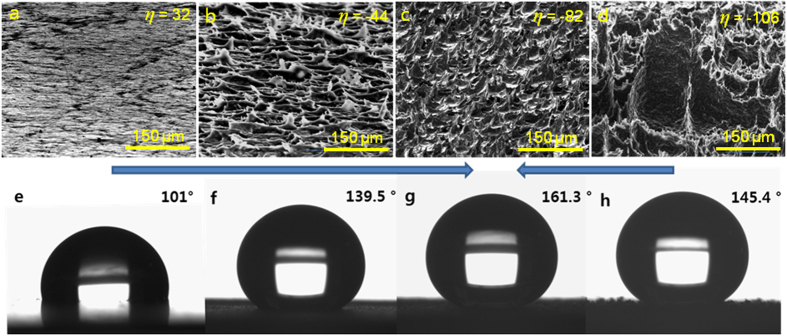
Surface morphology and contact angle image of the patterns produced at different shear rates. Tilted SEM images of films fabricated at (**a**) *η* = 32 s^–1^, (**b**) *η* = −44 s^–1^, (**c**) *η* = −82 s^–1^, and (**d**) *η* = −106 s^–1^. The corresponding WCA behaviors are in (**e**–**h**).

For a given film thickness and viscosity, we could optimize the pattern of the SH surface. The surface topology could be controlled through the effective shear rate *η* = Δ*V*/*h* between the two rolls, where Δ*V* = *V*_*1*_ *−* *V*_*2*_ and *h* is the thickness of film. A smooth film without any patterning was formed when *η* > 0 s^−1^ (*e.g.*, for *η* = 32 s^−1^, *V*_1_ > *V*_2_), as shown in Fig. 3a, and irregular surface morphologies were observed when *η* < 0 s^−1^, as shown in Fig. 3b–d. At a certain shear rate (*e.g.*, for *η* = −82 s^−1^, *V*_1_ < *V*_2_), a uniform SH shark-skin–like pattern developed, as shown in Fig. 3c,g. The pattern morphology possesses larger features beyond the optimal shear rate (*η* > −82 s^−1^) for SH surface, resulting in a loss of hydrophobicity, as shown in Fig. 3d,h.

Figure 2c showed the relationship between the shear rate between the two rolls and the WCA. When *V*_*2*_ is larger than *V*_*1*_, WCA is gradually increased up to 161.3° at a shear rate of *η* = −82 s^−1^, while hydrophobicity is decreased after *η* *<* −82 s^−1^. The sliding angle (SA) is another important parameter describing SH surfaces and can be measured as the angle where the droplet rolls off when tilting the substrate. With similar trend of WCA, minimum SA (below 5°) is observed at a shear rate of *η* = −82 s^−1^ indicating a self-cleaning surface as shown Fig. 2c. In addition, we measured the advancing angle, 155°, and the receding angle, 150° at a shear rate of *η* = −82 s^−1^ by dynamic sessile drop method. The hysteresis was around 5°. The temporal stability of the liquid contact angle on the SH surface was shown to the supplementary information. (Supplementary Fig. 3) Lager negative *η* values (*i.e.*, *V*_1_ < *V*_2_) increased the surface roughness, as shown in Fig. 2d. At even higher shear rates (*i.e.*, with *V*_1_ ≪ *V*_2_), the entire film was transferred from roll #1 to roll #2 observing starvation of the paste on roll #1.

### Mechanism underlying the formation of a bioinspired SH surface

Instability in Newtonian or non-Newtonian flow was typically regarded as having a negative impact on roll-coating processes for fabricating uniform films due to ribbing or zigzaging^26^,^27^,^28^,^29^,^30^. However, in our work we have exploited such characteristics to pattern the SH surface. A meniscus forms in the fluid region where two neighboring rollers separate, and the fluid runs on the rollers as shown in Fig. 1b. Instability in the meniscus occurs when the shear force and surface tension are not balanced. While the fingering instability of Newtonian fluids has been investigated analytically and experimentally^26^,^27^,^28^,^29^,^38^,^39^, there have been few studies on the related instabilities for *non-*Newtonian flows, for liquids with extremely high viscosity, as for the paste used in our study. An apparent morphological change from flat to rough pattern (Fig. 3a,b) was observed at a shear rate between 32 s^−1^ and −44 s^−1^. It is claimed that the creation of rough surface pattern is directly related to the sign change in *V*_*1*_ − *V*_*2*_, that is, *V*_*1*_ − *V*_*2*_ > 0 in Fig. 3a, whereas *V*_*1*_ − *V*_*2*_ < 0 in Fig. 3b–d. According to Grillet’s study^28^, tree-like structures were observed in eccentric cylinder forward roll coating flow for both Newtonian and non-Newtonian fluids, while not in the eccentric roll-and-plate flow. The speed of the outside roll (say *V*_*2*_) is higher than that of the inside roll (say *V*_*1*_) in the eccentric forward roll coating (*V*_*1*_ − *V*_*2*_ < 0), while the speed of the outside roll is less than that of the inside roll in the roll-and-plate coating (*V*_*1*_ − *V*_*2*_ > 0).

**Figure 4:**
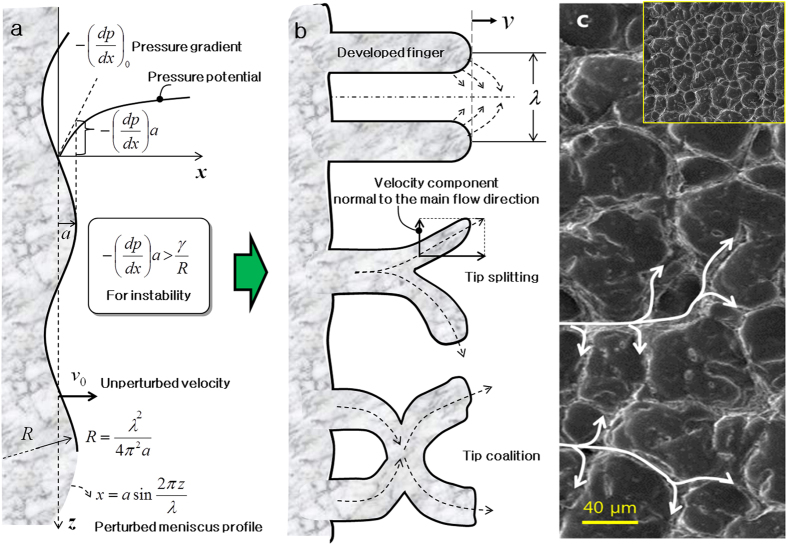
Formation mechanism of the shark-skin–like pattern. (**a**) Schematic diagram showing the relationship between the pressure gradient at the advancing front and surface energy^26^. (**b**) Schematic of the velocity and pressure gradient component radiating outward in the *z*-direction, tip splitting, and tip coalescence. (**c**) Top-view SEM images of the patterned surface fabricated at η = −82 s^−1^. Inset indicates low resolution image.

We indicate that fingering instabilities associated with non-Newtonian flows between the rollers are necessary for the formation of the observed rough patterns as shown in Fig. 4^26^,^27^,^28^,^29^,^38^,^39^. The flow velocity (*v*_0_) is proportional to the pressure gradient (*dp*/*dx*)_0_ at the meniscus front, and the curvature of the front (*R*) and the surface energy (*γ*) determine the flow-surface geometry, as in Fig. 4a. The *R* = *λ*^*2*^/(4*π*^*2*^*a*) in the case where the meniscus is perturbed into a sinusoidal shape with amplitude *a* and wavelength *λ*. If the unperturbed velocity profile is assumed to be *v*_0_ = −(*h*^*2*^*/12μ*)·(*dp*/*dx*)_0_^26^, where μ is the shear viscosity of the paste, the front instability will occur under conditions where −*a*(*dp*/*dx*)_0_ > *γ* /*R*. With:




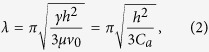




where *C*_*a*_ = |*μv*_0_/*γ*| is the capillary number and *v*_0_ = (*V*_*1*_ + *V*_*2*_)/2. The *C*_*a*_ describes the relative influences of the viscous and surface forces in the instability region, where the former causes the instability, while the latter acts as a stabilizer. The velocity of each roll, the effective shear rate and *C*_*a*_ produced between the two rolls are summarized in Supplementary Table 1.

Under optimal conditions where WCA reaches the maximum in Fig. 3c, *C*_*a*_ was very high^26^,^27^,^28^,^29^,^38^,^39^ at ~ 2700, from the values in Fig. 2b (μ = 658 Pa·s) and in Supplementary Table 1. Here, *v*_0_ = 4.1 cm/s, where *v*_0_ was estimated from the speeds of the two rollers, (*V*_*1*_ + *V*_*2*_)/2 (=*V*_*avg*_), and a *γ*- value of 21.8 mNm^−1^ for uncured MWCNT paste film, as determined using the Owens-Wendt method^40^. Such a high *C*_*a*_ in our study was primarily attributed to the high viscosity of MWCNT paste. The measured microstructure sizes (*λ*) reached around 60μm from top view of the pattern images in Fig. 4c and Supplementary Fig. 2e. The average height of the SH structures was 30 ~ 40 μm (cf. Supplementary Fig. 2a). The calculated microstructure sizes (*λ*) from equation (2) was 17.4 μm, close to the size observed in the actual surfaces. In addition, the expectation from the equation (2) that the increase in the pattern size with decreasing *C*_*a*_ was consistent with our observation as shown in Fig. 3 and Supplementary Fig. 4.

The relative error between the observation and the calculation of *λ* from Equation 2 was believed to result from the assumption that the velocity was established under incompressible and Newtonian flow conditions. There may be significant differences between the velocity profile modeled for Equation (2) and that of the non-Newtonian MWCNT/PDMS paste by shear-thinning phenomenon^35^. In addition, velocity and pressure components in spanwise direction of the rolls in Fig. 4a,b increase as the capillary number (or the viscosity) increases^26^,^28^,^29^. As the flow velocity or the capillary parameter increase, the fingering structures grow parallel to circumferential direction of the roller and eventually to neighboring fingers, and then form branched or tree-like structures^26^,^28^,^29^. The tip-splitting can be treated as signature of the velocity components radiating outward to the spanwise direction of the rollers due to the pressure gradients as shown in Fig. 4b,c. However, the velocity profile has been assumed to have a component in the circumferential direction only.

### Durability performance of the SH films

**Figure 5:**
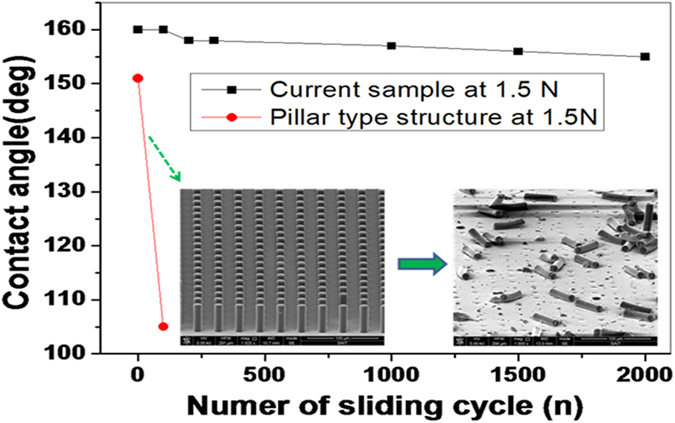
Mechanical durability of the SH surfaces. WCA values of the shark-skin–like pattern and a pillar-type pattern after *N* wear-test cycles by a rubber tip under an applied normal load of 1.5 N. The pillar-type surfaces, shown inset, are severely damaged after 100 cycles and show significant degradation of hydrophobicity. The shark-skin–like surface maintains its contact angle and morphology after 2000 cycles.

To evaluate wear durability for practical usage, a rubber tip under an applied normal load (of ~1.5 N) was dragged horizontally on the fabricated SH surface and on a pillar-type patterned surface (also see Supplementary Fig. 5). As shown in Fig. 5, the latter patterns were found to be severely damaged after 100 cycles, resulting in a considerable degradation of hydrophobicity (inset of Fig. 5). The produced rough/shark-skin–like pattern, on the other hand, maintained its morphology and hydrophobicity even after 2000 cycles, demonstrating its robustness to tribological/physical damage^16^,^25^,^41^.

### Drag reduction performance of the SH film

**Figure 6:**
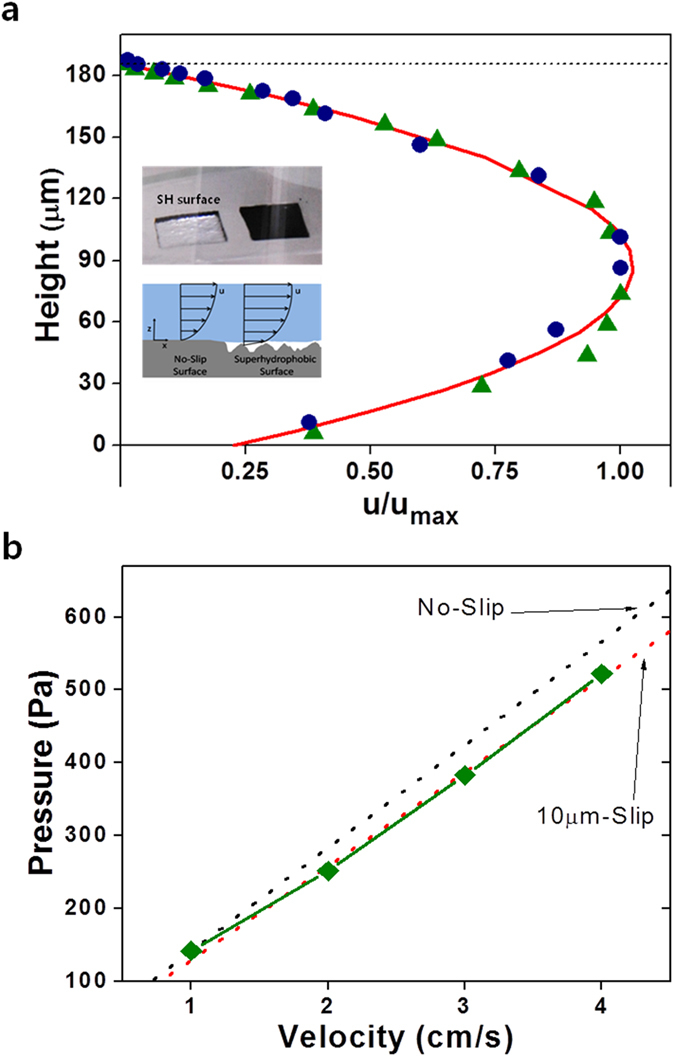
(**a**) Water velocity profiles for water flow on SH patterned surfaces and (**b**) Pressure drop through a channel in accordance with the velocity measurements. The lower surface is SH while the upper surface is a glass coverslip. Data points (▴, •) correspond to the fluid velocity averaged over the field of view at each height during two experimental runs under the same conditions. The solid line corresponds to a fitted pressure-driven Poiseuille flow profile with a slip boundary condition applied to the lower surface. Upper inset shows silvery mirror-like reflection phenomenon when submerging the SH film under water at certain tilting degrees. Lower inset compares schematically the velocity profiles under standard *no-slip* conditions and that over air filled pockets. The pressure was obtained from a 12 cm long channel with a height of 300 um tested at four velocities. The figure shows the pressure drop that would be expected with a smooth channel and one with a 10 μm slip on the lower surface.

Generally, the relative velocity at the boundary between a solid wall and a liquid is considered to be zero, through the widely accepted “no-slip” boundary condition. However, for the case of SH surfaces, the fluid velocity near the solid surface appears to exhibit a non-zero velocity when averaged over the complete surface, taking into account the air/fluid areas, i.e., induced through a layer of air that can form in the interstices of the rough surface (inset scheme of Fig. 6), which acts as a shear-reducing boundary between the solid surface and the fluid, greatly reducing the drag^42^,^43^.

In the fabricated SH film case, a mirror-like surface was observed when submerging the SH film under water at certain tilting degrees as shown inset of Fig. 6 (also see Supplementary Fig. 6). This reflection occurs due to the presence of fully covered air layer between the SH films and water interface^44^.

To evaluate the impact of the SH surface, the velocity profile of the fluid flowing over the surface was obtained using micro-Particle Imaging Velocimetry (μ-PIV) techniques (see Supplementary Fig. 6)^45^. A fluid channel was constructed that incorporated the SH surface as the lower wall and a glass coverslip as the upper wall. The average velocity of the fluid in laminar flow conditions (with a Reynolds number, *Re* of close to 1) was obtained across the height of the channel. The experimentally investigated conditions correspond to pressure driven Poiseuille flow conditions. The velocity profile in Fig. 6 shows the results from the surface with features of height of 30 ~ 40 μm and an RMS roughness of 9 μm, corresponding to a surface akin to that of Fig. 3(c). It was clearly seen that at the upper glass coverslip surface, the velocity tends to zero, corresponding to the expected non-slip condition. However, the velocity on the SH surface (set as the air-water interface) was non-zero (u/u_max_ = 0.23 from the fitted curve), with a corresponding slip length of 13μm (from Navier’s boundary condition, V_s_ = λ ∂u/∂y). Note that the implication of the slip length was that the liquid may be considered stagnant only at 13μm *into* the SH surface. The reported slip length value was one of the largest in literature^43^. It was then concluded that fluid flow slip does occur considerably at the SH surface, with concomitantly robust drag reducing characteristics. A more detailed interpretation of the drag reduction effects of the synthesized SH surfaces, such as their variation with *Re*, mean surface roughness, and lateral length scale as well as measurement of the pressure drop reduction in channel flow, is presently under investigation.

In addition to the velocity profile measurements, and recognizing the uncertainty in determining the surface location for rough unpatterned surfaces, the pressure drop was measured across a larger microchannel, 12cm long by 300 μm high. The pressure drop results are shown in Fig. 6. The dotted lines show the pressure drop for a smooth microchannel and for one with a 10 μm slip on the lower surface. The pressure drop results shows the response is close to the 10 μm slip result.

## Discussion

In summary, the synthesis of a mechanically robust rough surface, which seems akin to that of a bristled shark skin, exhibiting SH characteristics, through a relatively inexpensive novel roll-to-roll process has been demonstrated. The synthesis technique is simple to set up, reproducible, amenable to industrial scale production as long as we use larger size rolls, and can be adapted to widespread usage. The underlying mechanism for the formation of the rough surface has been indicated to be fingering instabilities associated with high viscosity liquids subject to roll-to-roll processes. The characterization of SH surfaces was done through static means (*e.g.,* through contact angle measurements), as well as under dynamic/liquid flow conditions, where significant drag reduction through a pressure drop reduction was observed. The significant merit of our approach for large scale fabrication of SH surfaces is that nano-/micro-scopic patterning or chemical treatment is unnecessary for exhibiting superior SH characteristics. In addition, electrical and thermal properties may be tuned through desired electrically conductive fillers.

## Methods

### Raw material preparation and characterization

For the fabrication of the high viscosity carbon nanotube(CNT) paste, polydimethylsiloxane (PDMS, Sylgard 184 silicone elastomer base) was purchased from Dow-Corning (Midland, MI, USA), and multiwalled carbon nanotubes (MWCNTs) with an outer diameters of 10–20 nm and a lengths of 100–200 μm were purchased from Hanwa Nanotech, Inc. (Seoul, Korea). To ensure effective mixing and dispersion of the highly entangled CNTs within the polymer matrix, the pastes were premixed using a paste mixer and then a three-roll mill. MWCNTs and the PDMS base elastomer were combined in various weight ratios using the following procedure: First, the elastomer base and curing agent were mixed in a weight ratio of 10:1. This mixture was then combined with the MWCNTs in the paste mixer for 1 min. The resulting CNT pastes were milled for several minutes while gradually decreasing the gap between the rolls. The dynamic viscosity of the pastes was measured using a rheometer. Rheometry was conducted at room temperature in a small-amplitude oscillatory shear mode using a 20 mm parallel plate geometry with a frequency sweep from 10^−1^ rads^−1^ to 10^2^ rads^−1^.

### Pattern fabrication

A double-roll machine was constructed to fabricate the random SH film surfaces (Supplementary Fig. 1). The diameter and the length of the rolls were 30 mm and 300 mm, respectively, and their angular velocities were adjustable independently up to 300 rpm. A polyimide tube was placed on roller #1 as a coating target material for the CNT paste film (Supplementary Figure 1(a)). When the velocity of roll #1 (*V*_1_) was much higher than the velocity of roll #2 (*V*_2_), flat surface films were deposited on roll #1 when the CNT paste was added between the two rollers (Supplementary Fig. 1(b),c)). At this point, inversion of the roller speeds to *V*_1_ < *V*_2_ induced capillary bridging between roll #1 and roll #2, followed by the eventual separation of the capillary bridges from roll #2 (*cf.* Fig. 1(b)). The optimal rolling speed ratio of roll #1 and roll #2 for generating the SH pattern depended on the viscosity of the CNT paste, the desired thickness of the sample, and so on.

### Film characterization

The morphologies of the patterned films were characterized using optical microscopy and scanning electron microscopy (SEM, Quanta field emission SEM, 650 FEG). The water contact angle (WCA) and sliding angle (SA) were acquired using a contact angle measuring instrument with a drop-shape analysis system (Drop shape analyzer, KRUSS). The volume of the deionized water droplets was around 7 μl. The average contact angles were determined from a minimum of five different locations. The SA was measured at the point just before roll-off when the sample on the stage was tilted. To investigate the durability of the patterned composite films, a wear tester was constructed. Briefly, a rubber (glass) tip with a radius of 2.5 mm was applied to the film surface at a normal load of 1.5N and dragged horizontally with a sliding speed of 25 mm/s. Following the wear test, WCA measurements and SEM images were used to re-characterize the surface properties.

### Velocimetry measurement

To measure the velocity profile, micro-PIV techniques were used. Fluorescent microspheres (polystyrene, diameter 1.0 μm, Bangs Laboratories, Inc) were distributed in distilled water and sonicated to ensure uniform particle dispersion. The flow was driven using a syringe pump through a channel (height: ~200 μm, width 9mm) at a centerline velocity of ~0.5 mm s^−1^, corresponding to flow in the laminar regime, Reynolds number (*Re*) of the order of 1, as shown Supplementary Fig. 6. The fluid was imaged under magnification starting below the lower SH surface and past the upper non-SH surface. Upper surface can be determined by the presence of in-focus, stationary, adsorbed particles on the glass coverslip, as can the fluid/SH surface interface locations, however, due to the presence of air pockets throughout, the lower surface was set at the fluid/air/surface interface height. A set of 5 images spaced 30 ms apart were taken at each level, processed to remove background noise and out-of-focus particles, and used to generate an average velocity vector for the interrogation window (field of view) per height plane. The pressure was obtained from a 12 cm long channel with a height of 300um tested at four velocities.

## Additional Information

**How to cite this article**: Park, S.-H. *et al.* Bioinspired superhydrophobic surfaces, fabricated through simple and scalable roll-to-roll processing. *Sci. Rep.* **5**, 15430; doi: 10.1038/srep15430 (2015).

## Supplementary Material

Supplementary Information
