# Supplementary material for: Bioinspired superhydrophobic surfaces, fabricated through simple and scalable roll-to-roll processing
Source: Sci Rep. 2015 Oct 22;5:15430. doi: 10.1038/srep15430 (PMC4651109; doi:10.1038/srep15430)
Supplement: Supplementary Information [file srep15430-s1.pdf]

Supplementary Information for:

Bioinspired superhydrophobic surfaces, fabricated through simple and scalable roll-to-roll processing

Sung-Hoon Park[1]†,*, Sangeui Lee[2]†, David Moreira[3], Prabhakar R. Bandaru[3], InTaek Han[2], & Dong-Jin Yun[2]*

[1]*Department of Mechanical engineering, Soongsil university, 369 Sangdo-ro, Dongjak-gu, Seoul, 156-743, Korea*

[2]*Material Research Center, Samsung Advanced Institute of Technology, Yongin-si, Gyeonggi-do, 446-712, Korea*

[3]*Department of Mechanical & Aerospace Engineering, University of California, San Diego, La Jolla, CA 92093-0411, USA*

†These authors contributed equally to this work.

*Email: leopark@ssu.ac.kr (S.-H. Park), dongjin.yun@samsung.com (D.-J. Yun)

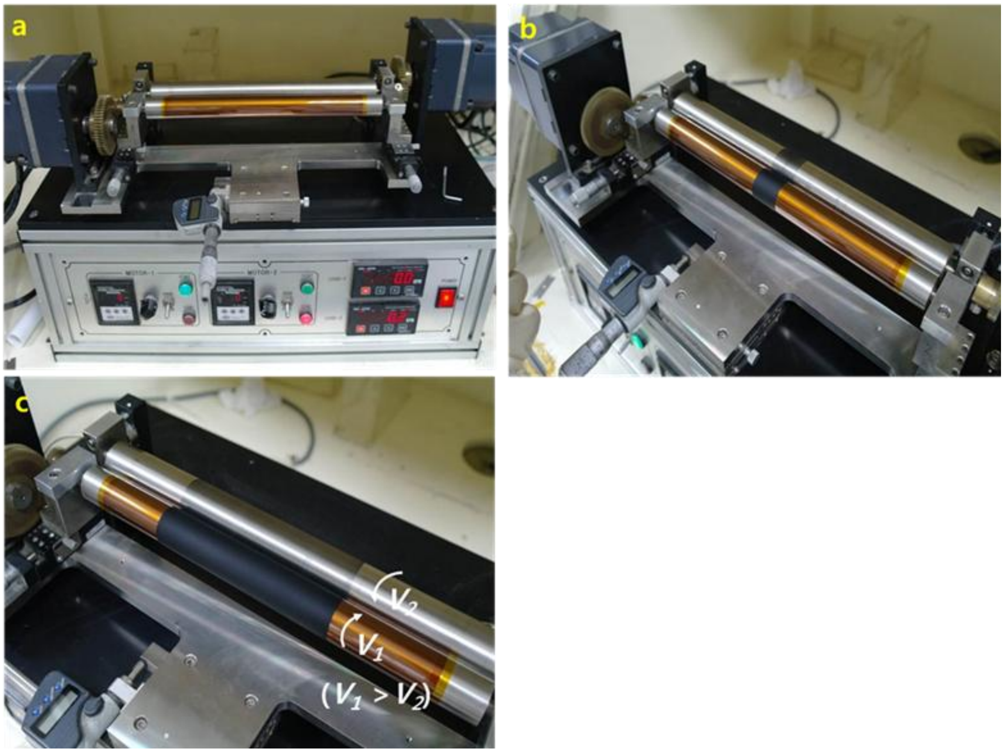

Supplementary Figure 1. The film formation process.

(a) The double-roll film machine built to fabricate the thin films on the substrates. (b), (c) When a viscous paste (MWCNT paste) is placed in the nip between the rolls, all of the material is transferred onto the roll with the higher speed.

To make the SH pattern surface, we need to regulate the relative velocities of the two rollers, for a given material viscosity and roll geometry (ex. distance), and can be accurately done. Through double-roll film machine above, we can control each roll speed precisely. And certain shear rate between two roll, the SH pattern is fabricated.

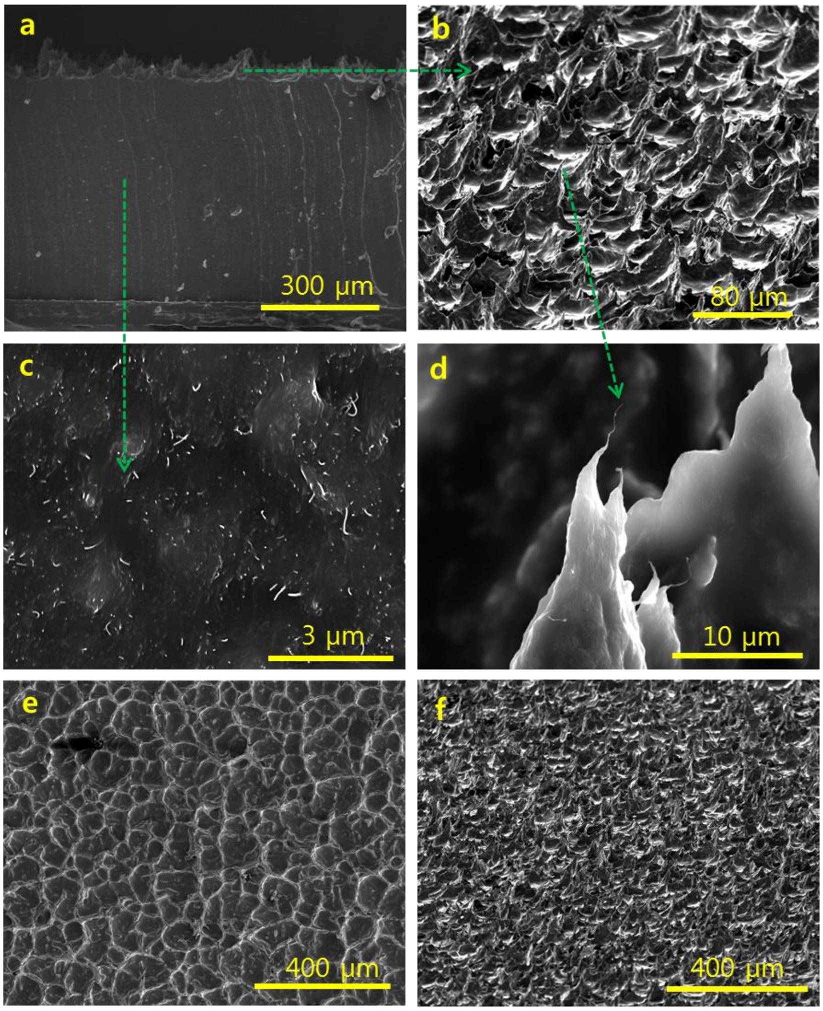

Supplementary Figure 2. Structure of the shark-skin–like pattern.

(a) SEM image of the cross-section of the patterned film. (b) SEM image of the surface structures, tilted to show the texture. (c) SEM image of the bulk 10 wt.% MWCNT composite film produced with uniform dispersion conditions. (d) High-resolution SEM image of an individual pattern. (e) Top-view and (f) tilted view of the patterned film at lower magnification.

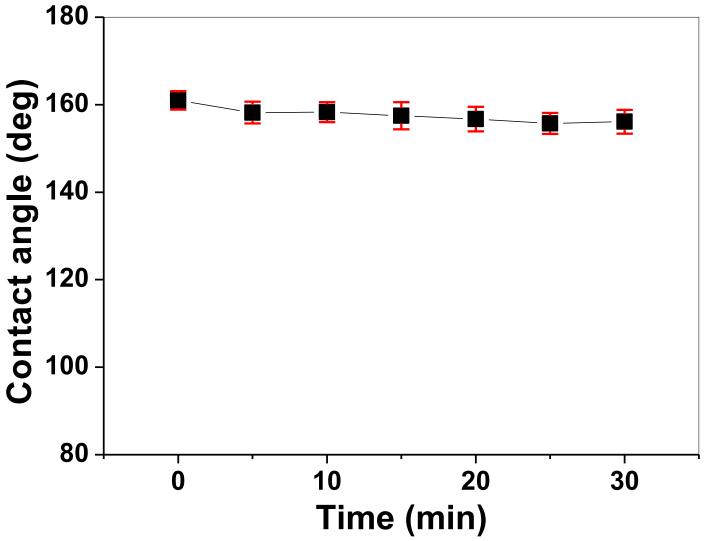

Supplementary Figure 3. Temporal stability of the liquid contact angle on the SH surface.

The water contact angle on the patterned surface with the initial water droplet within 30 minutes is shown in the graph. It was seen that the contact angle was constant over 30 minutes, indicating good stability. (at room temperature)

V_1 [cm s^{-1}]	V_2 [cm s^{-1}]	V_2 / V_1	**Shear rate ($\eta = \Delta V / h$)** [s^{-1}]	**Viscosity [Pa·s]**	**C_a**
12.6	11	0.9	32	1570	8498
8.8	11	1.0	-44	1170	5313
6.9	11	1.6	-82	658	2701
5.7	11	1.9	-106	519	1988

Supplementary Table 1. The velocities of the rolls in each experiment and the associated effective shear rates

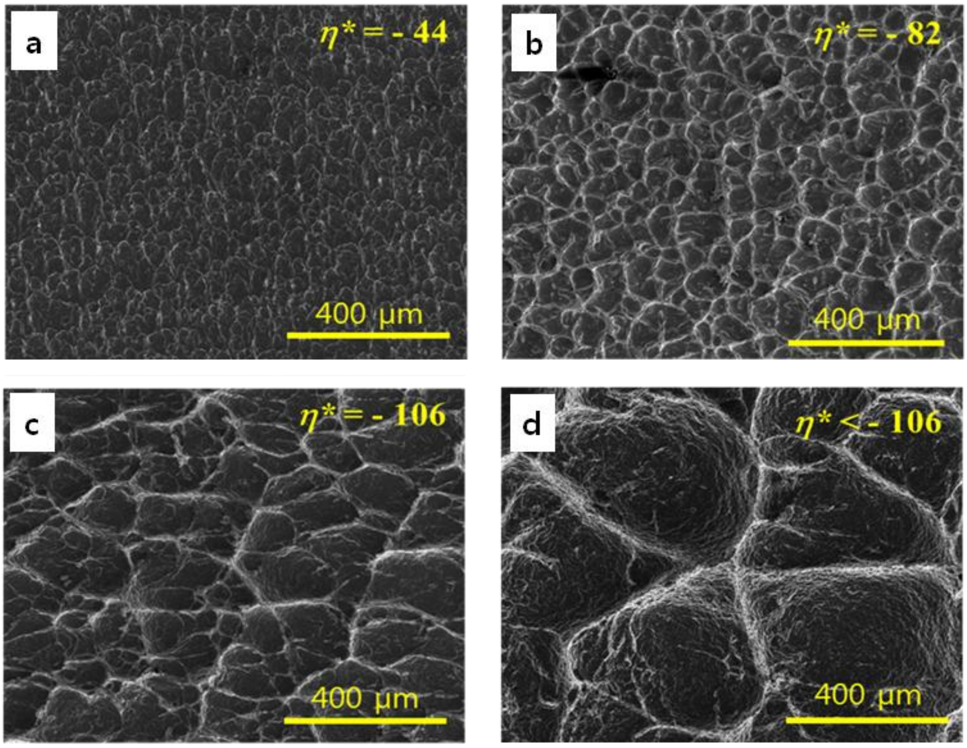

Supplementary Figure 4. Top-view SEM images of the developed films with varying shear rate. (a) η = –44 s^{-1}, (b) η = –82 s^{-1}, (c) η = –106 s^{-1}, and (d) η < –106 s^{-1}.

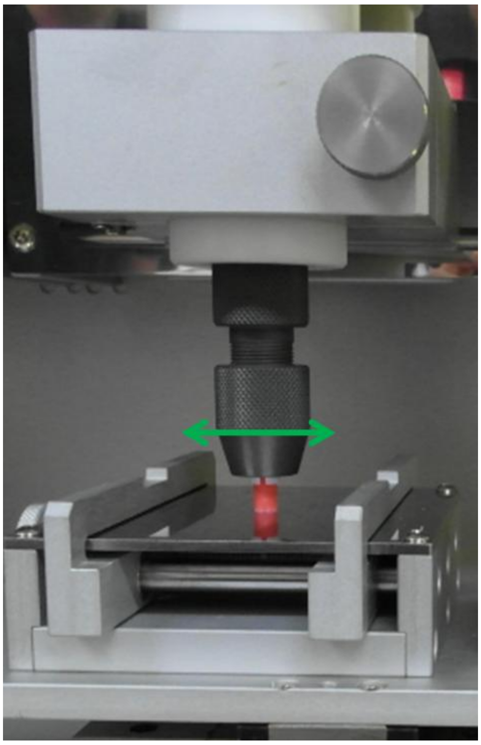

Supplementary Figure 5. Wear testing of the patterned films.

A rubber or glass tip with a radius of 2.5 mm under the desired normal load (*e.g.* 1.5 N) was dragged horizontally with a sliding speed of 25 mm s^{-1}.

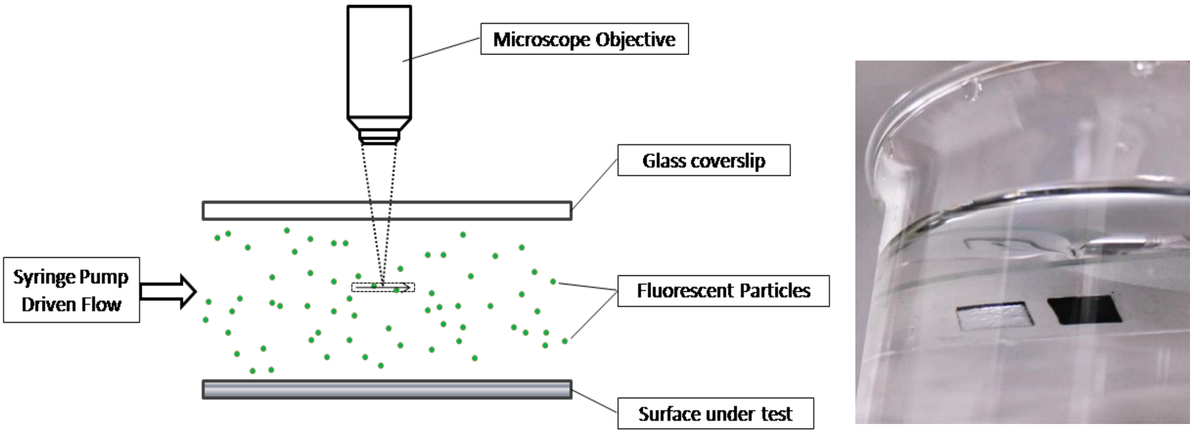

Supplementary Figure 6. PIV measurements and images showing air layer (light color) on the SH surface compared to non-SH surface.
